# Longer-Term Outcomes of the Incredible Years Parenting Intervention

**DOI:** 10.1007/s11121-020-01176-6

**Published:** 2020-10-27

**Authors:** Geertjan Overbeek, Jolien van Aar, Bram Orobio de Castro, Walter Matthys, Joyce Weeland, Rabia R. Chhangur, Patty Leijten

**Affiliations:** 1 Research Institute Child Development and Education, University of Amsterdam, Amsterdam, The Netherlands; 2 Utrecht University, Utrecht, The Netherlands

**Keywords:** Incredible years, Parenting intervention, Longer-term effects, Conduct problems, Broader benefits, Multi-informant

## Abstract

Conduct problems can develop into behavior disorders and put children at risk for other mental health problems. Parenting interventions have been shown to successfully reduce conduct problems and are often expected to prevent the development of broader mental health problems. Few studies have evaluated the longer-term and broader effects of these interventions. To what extent are parenting intervention effects sustained in the years after the intervention? And do effects pertain to conduct problems specifically, or do they also affect broader aspects of children’s mental health? We used a randomized controlled trial to assess the longer-term (2.5 years) effects of the Incredible Years parenting intervention on children’s conduct problems in an indicated prevention setting (*N* = 387; 79% retention rate). Using a multi-method (survey and computerized tasks) and multi-informant (parents, teachers, and children) approach, we tested whether initial effects on conduct problems were sustained, and whether Incredible Years had broader effects on children’s peer problems, emotional problems, attention-deficit/hyperactivity disorder (ADHD) symptoms, attention and inhibition deficits, and service use. Incredible Years, relative to control (no intervention), led to sustained reductions in parent-reported conduct problems (Cohen’s *d* = 0.31), but not teacher- and child-reported conduct problems. There were no broader benefits: Incredible Years did not reduce children’s peer problems, emotional problems, ADHD-symptoms, attention and inhibition deficits, or their service use. Improvements in parents’ perceptions of child conduct problems sustained until 2.5 years later. Our findings do not show benefits of Incredible Years as a preventive intervention for children’s broader mental health.

**Electronic Supplementary Information:**

The online version contains supplementary material available at 10.1007/s11121-020-01176-6.

Parenting interventions are the key strategy to reduce early conduct problems in children and are often expected to prevent behavior disorders and broader developmental difficulties later in life (Weisz and Kazdin 2017). Numerous meta-analyses show the beneficial effects of parenting interventions on conduct problems immediately after intervention (e.g., Michelson et al. 2013; Piquero et al. 2016). Much less is known about how well these effects sustain over a longer period, and how well they prevent broader mental health problems, such as emotional problems (e.g., feelings of sadness and anxiety) and peer problems (e.g., bullying perpetration or victimization). To illustrate, recent reviews show that only about 10% of the randomized parenting intervention trials follow children from both the intervention and control condition longer than 1 year after the intervention (Leijten et al. 2018). Moreover, only half of these longer-term trials included at least one broader measure of children’s mental health, such as emotional problems. Yet, knowing the longer-term effects of parenting interventions for a range of mental health outcomes is vital to understand the precise potential of parenting interventions to improve children’s lives. Using 2.5-year follow-up questionnaire data from parents, teachers, and children, and neuropsychological tasks, we tested the sustained and broader effects of the Incredible Years parenting intervention in an indicated prevention setting—for children with elevated levels of conduct problems.

## The Incredible Years Parenting Intervention

Early conduct problems include a spectrum of externalizing, antisocial, and rule-breaking behaviors, such as temper tantrums, arguing, and non-compliance. Although many young children occasionally show such behavior, some children show them more frequently and persist to show these behaviors across developmental phases (Bongers et al. 2003; Matthys and Lochman 2017). It may be hard for parents to redirect these behaviors and to avoid falling into a negative interaction pattern in which they unwillingly reinforce externalizing, antisocial, and rule-breaking behaviors (coercive cycles; Patterson 1982). Without effective family support, conduct problems in early childhood can therefore develop into disruptive behavior disorders (Loeber et al. 2000; Hoeve et al. 2008). These disorders in turn increase risk for academic problems, substance abuse, deviant peer associations, and delinquency (Capaldi 1992; Patterson et al. 2000).

The Incredible Years parenting intervention aims to prevent these unfavorable trajectories by reducing conduct problems in early childhood (Webster-Stratton 2001). It is an established behavioral parenting intervention which demonstrated immediate effects in numerous trials (see Menting et al. 2013, for a meta-analysis). During 14 weekly group sessions, parents learn relationship building techniques (e.g., play and involvement), positive reinforcement techniques (e.g., praise and celebrations), and non-violent discipline techniques (e.g., limit setting and ignore). With these techniques, parents are guided to shift their attention from negative to positive child behavior, thereby avoiding coercive interactions and increasing positive interactions. Incredible Years is recommended by the Dutch (NJI) and British (NICE) registry of evidence-based interventions but endorsed as “promising” rather than as “effective” in the USA (Blueprints) due to a lack of high-quality longer-term assessments (> 1-year follow-up).

We conducted a longer-term follow-up of the effects of Incredible Years for reducing conduct problems in a prevention setting with children who scored at or above the 75th percentile on the Eyberg Child Behavior Inventory (ORCHIDS; Chhangur et al. 2012). Immediately after the intervention and 4 months later, families showed reduced parent-reported, but not observed, conduct problems, parallel with improvements in parent-reported and observed parenting practices (Weeland et al. 2017; Weeland et al. 2018a). In this paper, we present the effects of Incredible Years on children’s broader mental health 2.5-year post-intervention.

### Are there Sustained Effects on Conduct Problems?

On the one hand, the parent-perceived effects of parenting interventions such as Incredible Years may sustain. If children’s conduct problems reduce when parents participate in Incredible Years, this will probably reinforce parents’ more positive strategies, reducing conduct problems further. Related to this, when parents see that their parenting strategies reduce children’s conduct problems, feelings of parental competence and satisfaction may increase (Levac et al. 2008). In turn, this may help them keep using these strategies, also in the face of new challenges (Bandura 1977; Mouton and Roskam 2015; Deković et al. 2010).

On the other hand, the parent-perceived effects of parenting interventions such as Incredible Years may not sustain. As for any new behavior, it might be hard for parents to stay positive and use their newly learned strategies consistently in the face of new challenges and absence of therapist support. Children with conduct problems frequently challenge their parents, and conduct problems tend to be fairly persistent (Shaw et al. 2005). If parents occasionally invest less in positive interaction or give in to their child’s challenging behavior, this could put the family at risk for falling back to coercive interaction patterns. Empirically, systematic reviews and meta-analyses suggest that, across different programs, parenting intervention effects on children’s conduct problems are sustained in the longer term (Lundahl et al. 2006; Sandler et al. 2011; Sandler et al. 2015; Van Aar et al. 2017).

For the Incredible Years program specifically, randomized trials with a controlled follow-up 1 year after the intervention report mixed findings. Some show that initial effects on conduct problems were retained (Perrin et al. 2014; Brotman et al. 2008; Gross et al. 2003; Kim et al. 2008; Lavigne et al. 2008), while others show that initial effects had faded out (Reedtz et al. 2011; Stewart-Brown et al. 2004). What explains this variation remains unknown.

The only longer-term randomized controlled trial of Incredible Years (> 1 year, including follow-up data of control group) included assessments 5 to 10-year post-intervention (Scott et al. 2014). Results suggested sustained improvements in conduct problems for children in a clinical setting, but not for children in a prevention setting. This might in part be because effect sizes tend to be smaller in prevention settings (Leijten et al. 2018). To be able to detect smaller effect sizes, larger sample sizes are needed. The sample size of 109 families in the prevention trial by Scott et al. (2014) may have been too small to detect such smaller prevention effects. Other follow-up studies have been reported, but due to waitlist control designs, follow-up data for control groups were not available. We conducted a longer-term (albeit shorter than Scott and colleagues) follow-up including the control group in a large randomized controlled trial (*N* = 387), to ensure sufficient power to detect smaller effects on children’s conduct problems.

### Are there Broader Benefits of Incredible Years on Children’s Development?

Without intervention, coercive interactions and conduct problems can evolve into additional problems across different developmental domains. This might be due to the psychological burden and social isolation or marginalization that children may experience because of the negative interactions with parents, other authority figures, and peers that result from their conduct problems (e.g., dual failure model; Capaldi 1992). More specifically, children’s conduct problems may lead to accumulating experiences of failure and rejection by parents and, if carried forward to the school context, by peers and teachers that may contribute to depressive feelings. Thus, the fact conduct problems often co-develop with other mental health problems (e.g., Overbeek et al. 2001; Beauchaine and McNulty 2013) raises the question whether a parenting intervention that reduces children’s conduct problems also prevents broader mental health problems, such as peer problems or emotional problems.

Previous research indicates that, indeed, parenting interventions may yield broader benefits in the longer term. In trials of the New Beginnings Program and Family Bereavement Program—aimed at teaching effective parenting skills, active listening skills, and behavior management strategies to increase positive family interactions—significant reductions in emotional problems and substance use were found at 6-year and 15-year follow-ups which, at the 6-year follow-up, coincided with significantly increased self-esteem, educational goals, and job aspirations. Such longer-term, broader benefits of parenting interventions may come about because the improvements in parenting cause an increase in children’s competencies and self-regulation abilities (see Sandler et al. 2011), which in the longer term may be related to a range of positive child development outcomes (Eisenberg et al. 2010).

For the Incredible Years program specifically, the evidence for broader parenting intervention effects is mixed. Some trials indeed suggest broader benefits of Incredible Years on emotional problems (e.g., Webster-Stratton and Herman 2008), but others did not find this (e.g., Stewart-Brown et al. 2004), and a recent individual participant data meta-analysis found no support for such broader benefits (Leijten et al. 2018). Although no clear-cut explanations can be presented to explain these mixed findings, they may have come about as consequence of differences between studies in sample size, measurements, and sociodemographic sample characteristics.

Importantly, estimating broader benefits requires well-powered longer-term assessments, because broader effects may be even smaller than effects on the targeted behavior and may need time to evolve before they become apparent. In addition, broader benefits are ideally assessed using multiple informants, because children’s peer problems are not always observable for parents (Winsler and Wallace 2002), nor are negative emotions and feelings (Sourander et al. 1999). Therefore, only longer-term assessments with multiple informants, including teachers and children themselves, can rigorously test whether Incredible Years has broader benefits on children’s peer problems and emotional problems.

In addition, many children with conduct problems show symptoms of attention-deficit/hyperactivity disorder (ADHD; prevalence of comorbidity is 24–33%, Waschbusch 2002). These symptoms include inattentive, impulsive, and hyperactive behaviors that are related to underlying neuropsychological deficits, such as sustained attention and inhibitory control (American Psychiatric Association 2013). In contrast to conduct problems, ADHD symptoms are less driven or maintained by parenting practices (Burke et al. 2008) and may therefore be less likely to change when parenting changes (hence, the development of more ADHD-specific programs, e.g., Sonuga-Barke et al. 2006). Although previous research has generally shown favorable effects of Incredible Years and other parenting interventions on parent-reported ADHD symptoms (Leijten et al. 2018), less is known about whether such effects speak to the core of the ADHD construct—assessed as children’s inhibitory control ability. Indeed, parenting interventions generally do not reduce ADHD symptoms as reported by blind assessors (Daley et al. 2014). Thus, in addition to reports of blind assessors and parents, neuropsychological tasks can provide information on whether parenting interventions could alleviate core deficits in sustained attention and inhibitory control.

Finally, it is important to know whether Incredible Years reduces parents’ and children’s service use. Conduct problems lead to increased societal costs, primarily because children with conduct problems need special education and mental healthcare more often than children without conduct problems (Gustavsson et al. 2011; Romeo et al. 2006). When children’s conduct problems reduce, their service use may reduce, because they may be less likely to drop out from regular schools, and their parents may be less likely to seek additional professional help regarding their child or childrearing. Indeed, one previous study demonstrated that receiving the Incredible Years parenting intervention was associated with less use of additional mental healthcare (Weeland et al. 2017). In this study, we will examine whether Incredible Years impacts families’ service use in the years after intervention (e.g., use of special education services, use of additional mental healthcare related to parenting or child behavior problems).

### The Present Study

Our aims are to test 2.5 years after the intervention: (1) whether Incredible Years effects on conduct problems were sustained and (2) whether Incredible Years had broader benefits for children’s peer problems, emotional problems, ADHD symptoms, ADHD-related neurocognitive deficits, and parents’ and children’s service use. Using a multi-method (questionnaire and neuropsychological tasks), and multi-informant (parents, teachers, and children) approach, we aim for a precise estimation of the magnitude and width of the longer-term preventive impact of an established, evidence-based parenting intervention on children’s mental health. Our aim was to identify immediate and sustained effects of Incredible Years across the longest (2.5 years) time interval available in our study, so as to provide a most stringent test of longer-term follow-up effects. This 2.5-year time interval places our study in the 7% of studies with longest post-intervention time intervals (18–36 months), as specified in a recent meta-analysis of parenting intervention follow-up effects (Van Aar et al. 2017).

## Method

### Procedure

This study is a follow-up of the ORCHIDS randomized controlled prevention trial preregistered protocol: Chhangur et al. 2012). For the original trial, families were screened and recruited through community records via two Dutch regional healthcare organizations. All families with children ages 4–8 years (*N* = 20,048) of four (i.e., two large and two small) municipalities received a personalized information letter, including a consent form and a screening questionnaire for conduct problems at or above the 75th percentile on the Eyberg Child Behavior Inventory (ECBI; Eyberg and Pincus 1999). Eligible families were invited to participate and, if they gave consent, randomized to either control (no intervention) or experimental condition (Incredible Years). One parent-child dyad per family was invited to participate. Parents of either sex and of any ethnic group (mastering the Dutch language) were eligible. They initially participated in three waves of assessment: a pretest; a post-test immediately after the intervention (i.e., 4 months after pretest procedure); and a follow-up 4 months after intervention (i.e., 8 months after pretest; Weeland et al. 2017; METC UMCU, protocol number 11–320/K). We added two more waves: a follow-up 1.5 years after intervention (i.e., 22 months after pretest) and 2.5 years after intervention (i.e., 34 months after pretest). Procedures of these follow-up assessments were approved by the research ethics committee of the University of Amsterdam (under record 2015-CDE-6392), and renewed written informed consent was obtained from participating families.

### Participants

Originally, 387 parent-child dyads participated (197 interventions; 190 controls). Parents (92% mothers) were between 23 and 51 years (*M =* 38.10, SD = 4.84); children (45% girls) were between 4 and 8 years at baseline (*M* = 6.31, SD = 1.33). Most parents (88%) and children (97%) were born in The Netherlands. About half of the parents completed a form of higher education (i.e., higher vocational training or university), and most parents (91%) were married or living together with a partner. About 28% (28.6%) of the participating families received additional mental health or family care (e.g., mental healthcare for parents or social services), and 8% of children used some form of psychoactive medication—mainly psychostimulants—between pretest and post-test. For further details regarding study procedures and sample characteristics, see Weeland et al. (2017).

**Fig. 1:**
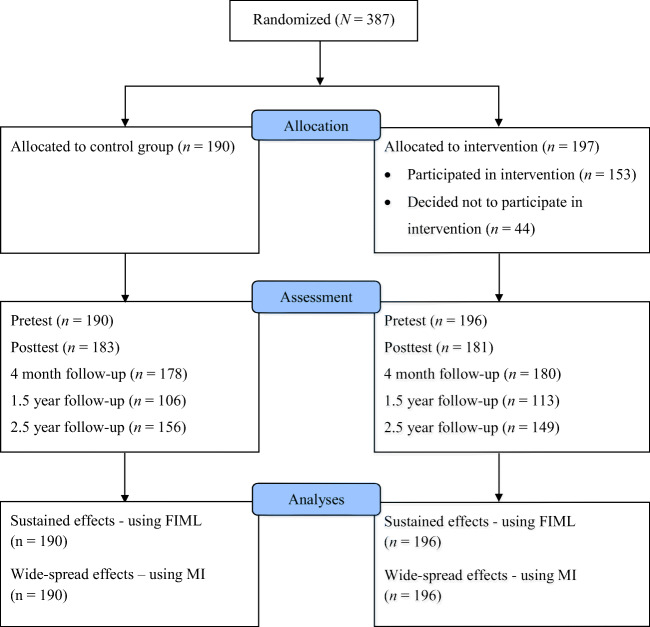
Flow diagram of participants in the study

At 2.5-year follow-up, 305 parent-child dyads participated (149 interventions; 156 controls). This is 79% of the parent-child dyads that participated at baseline (Fig. 1,^1^). Reasons for dropping out were lack of time to fill out the questionnaires, (upcoming) divorce of parents, or other personal circumstances. Families that dropped out did not differ from those who remained in the study on sociodemographic characteristics (e.g., child age and gender, ethnicity, educational level, single parenthood), baseline levels of negative parenting and child mental health, or assignment to condition (Table 1 of the online supplementary file). However, there was an intervention by attrition interaction effect on immediate post-test of conduct problems (*F*(1, 362) = 5.202, *p* = .023), showing that among those who dropped out of the study, post-test conduct problems were higher for families in the intervention group than control group. Among those who remained in the study, it was the opposite: post-test conduct problems were lower for families in the intervention group than control group.

### Intervention Fidelity

All Incredible Years intervention groups were led by two group leaders, of which at least one was an experienced and certified Incredible Years group leader. All main leaders had a background in clinical child psychology, had experience running IY groups before the study commenced, and were officially certified by The Incredible Years Inc. (see also Weeland et al. 2017). All group leaders engaged in protocolled group leader feedback and supervision trajectories. Protocol adherence, monitored by protocol checklists of session elements (e.g., brainstorms and role plays), was on average 86%.

### Intervention Participation

Regarding intervention participation, we examined parents’ attendance. This was important, as previous research has found significant associations between attendance and parenting intervention effectiveness (e.g., Kazdin and Wassell 1998; Prinz and Miller 1994). To boost parents’ attendance, childcare was arranged for parents who attended intervention sessions during office hours. Parents were compensated for travel costs when needed. Of the families randomized to the intervention, 44 did not attend any session. The other parents—the parents who attended at least one session—attended on average 11.01 (SD = 3.69) out of 15 sessions (with 74% attending at least 10 sessions). Families in the control condition did not receive Incredible Years but, if needed, were provided with information about professional healthcare. A previous analysis (Weeland et al. 2017) demonstrated that intervention dosage (i.e., how many sessions parents attended) did not moderate the Incredible Years intervention effect on child behavior outcomes.

### Measures

An overview of the timing of measurements is specified in Table 2 of the online supplementary materials.

#### Conduct Problems

At all five measurement waves, parents reported on conduct problems using the intensity scale of the ECBI (Eyberg and Pincus 1999). The Dutch version of this inventory showed good internal consistency, validity, and test-retest reliability (Abrahamse et al. 2015). The intensity scale consists of 36 items (e.g., “has temper tantrums”) that are answered on a 7-point scale (1 = *never* to 7 = *always*). Internal consistency was good at all time points (*α*s > .85).

In addition, at 2.5-year follow-up, parents, teachers, and children reported on conduct problems using the Strengths and Difficulties Questionnaire (SDQ; Goodman 2001). The SDQ has shown good validity and test-retest reliability (Stone et al. 2015). The conduct scale consists of five items (e.g., “often loses temper”) that are answered on a 3-point scale (0 = *not true* to 2 = *certainly true*). Internal consistency in our sample was *α*_parent_ = .63, *α*_teacher_ = .68, and *α*_child_ = .55, which is in line with other findings (e.g., Bourdon et al. 2005) and most probably a result from skewness and limited response categories rather than actual low reliability (Stone et al. 2015).

#### Emotional Problems, Peer Problems, and ADHD Symptoms

At 2.5-year follow-up, parents, teachers, and children all completed the emotional symptoms scale, peer problems scale, and the hyperactivity scale of the SDQ (Goodman 2001). Baseline measures of emotional problems, peer problems, and ADHD symptoms were only available from parents. Each scale consists of five items that are answered on a 3-point scale (0 = not true to 2 = certainly true). For example, the emotional symptoms scale asks whether the child is “often unhappy, depressed or tearful”; the peer problems scale whether the child is “rather solitary, prefers to play alone”; and the hyperactivity scale whether the child is “easily distracted, concentration wanders.” Internal consistency at follow-up ranged from *α* = .65 to *α* = .76 for the emotional symptoms scale, *α* = .47 to *α* = .67 for the peer problems scale, and *α* = .71 to *α* = .87 for the hyperactivity scale, with lowest consistencies for child reports.

#### Neurocognitive Deficits Related to ADHD

At 2.5-year follow-up, children showed their response inhibition skills and attention span using the stop signal task (Verbruggen and Logan 2008). In this computerized neuropsychological task, children performed a choice reaction task (i.e., they had to indicate whether an arrow pointed to the left or right). On a random selection of the trials, an auditory stop signal instructed children to withhold their response. The mean time needed to withhold their response (indicative of the ability to suppress a response) reflected children’s inhibitory control. In addition, the mean reaction time of response to go stimuli reflected children’s attention. In both cases, a faster reaction time indicated better inhibition and attention. The stop signal task has shown good reliability (e.g., Congdon et al. 2012). We used a 16-trial practice block, followed by three 64-trial blocks with a break scheduled in between each block.

#### Service Use

At 2.5-year follow-up, we asked participants: “Did you receive professional help between this assessment and the previous one?” Parents answered this question with yes or no. In case parents answered “yes,” the follow-up question was “What kind of professional help did you receive?”. Parents could then mark a number of alternatives: (a) professional family help at home, (b) ambulatory professional help for your child at a mental health institute/child psychiatry institute/community health service institute/private practice (pedagogical or psychological help)/or parenting support institute, (c) institutionalized care for the child, or (d) other, such as financial help or individual therapy for the parents. In the present study, we dichotomized information across answer categories. The score on this service use variable thus represents the proportion of parents that had received some form of parenting-related or child mental health-related professional service. About half of the families in the intervention (51%) and control condition (53%) had received additional services.

### Analyses

To test for sustained effects, we conducted two analyses. First, we estimated the longer-term effect of Incredible Years on conduct problems (using the ECBI and the SDQ) at the final measurement wave, using analysis of covariance (ANCOVA), controlled for baseline levels of conduct problems. Second, we used LCM piecewise function in *M*plus to get a more in-depth view on changes in conduct problems during intervention and after intervention, by estimating the initial intervention effect and the putative sustained effect based on all five waves of ECBI data. This model has two slopes of development: one slope represents change in conduct problems during the intervention phase (i.e., pretest to immediate post-test), and one slope represents change in conduct problems during the follow-up phase (i.e., immediate post-test to 2.5-year follow-up). Model fit is considered good if the root mean square error of approximation (RMSEA) and standardized root mean square residual (SRMR) are < .05 and confirmatory fit index (CFI) value is > .95 (Hu and Bentler 1999). Previous analyses on the ORCHIDS data indicated that clustering effects were negligible and that IY intervention effects across the different groups were not dependent on therapists or therapist characteristics.

To test for broader benefits, we estimated the longer-term effect of Incredible Years on peer problems, emotional problems, ADHD symptoms and related neurocognitive attention and inhibition deficits, and service use at the final measurement wave, using separate ANOVAs in SPSS. Where baseline scores were available, we corrected for these (i.e., ANCOVA). Power to detect a small effect (*d* = 0.30) using ANOVA was 0.84. We calculated Cohen’s *d* to indicate the size of each significant effect based on between-group differences controlling for pretest levels of conduct problems (Eq. (8), Morris 2008), where *C*_*P*_ was a bias adjustment based on sample size (Eq. (10), Morris 2008):

$$ dppc={C}_p\left[\frac{\left({M}_{post,T}-{M}_{pre,T}\right)\left({M}_{post,C}-{M}_{pre,C}\right)}{SD_{pre}}\right] $$

Missing data were handled with full information maximum likelihood (FIML) for the piecewise model in *M*plus, the most powerful method for testing small effect sizes (Graham et al. 2007). As the ANOVAs were conducted in SPSS, which does not support FIML, missing data were handled with multiple imputation. This approach reaches equivalent power when the number of imputations exceeds the minimum of—in our case—twenty datasets (Graham et al. 2007). Therefore, twenty datasets were generated, and results were aggregated. To account for intervention by attrition effects on post-test conduct problems, conduct problems at each measurement wave were included as auxiliary variables. In addition, all analyses are based on the intention-to-treat principle, that is, including the 44 families in the intervention condition who did not participate any session of Incredible Years.

## Results

According to parents, 31% of the children in the intervention condition showed clinical levels of conduct problems before intervention (> 95th percentile on the ECBI; Weeland et al. 2018b) which reduced to 14% at immediate post-test and to 9% at 2.5-year follow-up. In the control condition, this percentage of children with clinical-level conduct problems reduced from 25 to 18% at immediate post-test and to 10% at 2.5-year follow-up. The percentage of children showing clinical recovery was significantly higher in the intervention condition between pretest and post-test (*χ*^2^ = 3.88, *p* = .049), and not significantly different between post-test and follow-up (*χ*^2^ = 1.15, *p* = .283). According to teachers, equal percentages (7%) of children in the intervention and control condition showed clinical levels of conduct problems at follow-up (> 95th percentile on the SDQ; Meltzer et al. 2000). On the self-report measure, 12% of the children in the intervention condition, and 16% in the control condition, reported clinical levels of conduct problems at follow-up (*χ*^2^ = 0.98, *p* = .323).

At pretest, children who showed more parent-reported conduct problems also had more parent-reported peer problems (*r* = .28), emotional problems (*r* = .25), and hyperactivity (*r* = .24, all *p*s < .001). Chi-square tests show that, compared to children with non-clinical levels of conduct problems, children with clinical levels of conduct problems were 2 times more likely to show clinical levels of peer problems, 2.5 times more likely to show clinical levels of emotional problems, and 3.5 times more likely to show clinical levels of hyperactivity (*p*s < .05).

### Are there Sustained Effects of Incredible Years on Conduct Problems?

At 2.5-year follow-up, parents who participated in Incredible Years reported reduced conduct problems compared to parents who did not receive Incredible Years on the ECBI (Table 2 of the online supplementary file). The effect size is small (*d* = 0.33) and comparable to the effect size at immediate follow-up (*d* = 0.35; see Table 3 of the online supplementary file). Teacher and child reports of conduct problems on the SDQ did not show significant differences between the intervention and control condition (Table 1).

**Table 1:** Means, standard deviations, and intervention effects at 2.5-year follow-up

	Incredible years mean (SD)	Control mean (SD)	*F/χ*^*2*^	*p* value^*^
Conduct problems (ECBI)				
Parent report^a^	114.23 (21.86)	116.94 (23.93)	4.69	**.031**
Conduct problems (SDQ)				
Parent report^a^	1.89 (1.66)	2.11 (1.82)	3.62	.058
Teacher report^b^	1.23 (1.34)	1.06 (1.40)	1.09	.299
Child report^b^	2.47 (1.65)	2.71 (1.89)	1.60	.207
Peer problems (SDQ)				
Parent report^a^	2.04 (1.87)	2.09 (1.87)	0.36	.551
Teacher report^b^	1.68 (1.54)	1.80 (1.73)	0.40	.528
Child report^b^	2.36 (1.63)	2.29 (1.71)	0.14	.713
Emotional symptoms (SDQ)				
Parent report^a^	3.54 (2.34)	3.45 (2.25)	0.01	.924
Teacher report^b^	2.35 (1.90)	2.36 (2.04)	0.00	.966
Child report^b^	3.23 (2.23)	3.19 (2.17)	0.03	.862
Hyperactivity (SDQ)				
Parent report^a^	5.12 (2.73)	5.43 (2.64)	1.23	.268
Teacher report^b^	4.29 (2.55)	3.93 (2.66)	1.66	.199
Child report^b^	5.06 (2.27)	5.29 (2.20)	0.93	.335
Inhibitory control (SST)^b^	323.59 (136.23)	320.97 (107.40)	3.36	.068
Attention (SST)^b^	661.72 (98.51)	656.75 (118.36)	0.05	.826
Service use^c^	51%	53%	0.10	.748

*ECBI* Eyberg Child Behavior Inventory, *SDQ* Strengths and Difficulties Questionnaire, *SST* stop signal test, performed by child

^a^ANCOVA, corrected for baseline values. ^b^ANOVA, no baseline values available. ^c^Chi-square test

^*^p values were not adjusted for multiple tests. With Bonferroni correction, the alpha level for significance would be *p* < .003

**Fig. 2:**
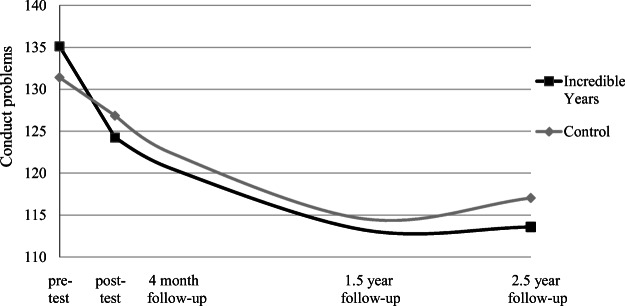
Estimated growth curves of the initial effect (pretest to post-test) and sustained effect (post-test to 2.5-year follow-up) of Incredible Years on parent-reported conduct problems (ECBI). The piecewise model showed that (1) the baseline levels of conduct problems were not significantly different between conditions, (2) the slopes from pretest to post-test were significantly different, and (3) the slopes from post-test to 2.5-year follow-up were not significantly different between intervention and control condition

The piecewise growth curve analysis showed that there was an initial effect of Incredible Years on parent-perceived conduct problems that were sustained until 2.5-year follow-up. The model showed that intervention condition (versus control condition) predicted the slope of conduct problems—measured with the ECBI—during the intervention phase, specifically, *B* = − 4.43, SE = 1.49, *p* = .003, and not the slope during follow-up phase *B* = − 0.07, SE = 0.32, *p* = .826 (model fit was good: RMSEA = .041, SRMR = .042, CFI = .992). This means that parents in the intervention condition reported a steeper decline in conduct problems during the intervention phase compared to parents in the control condition (i.e., 9.95 versus 5.52 points on the ECBI, *d* = 0.38) and equal levels of decline during the follow-up phase (i.e., both 4.37 points on the ECBI) so that parents in the intervention condition kept lower levels of conduct problems (see Fig. 2).

### Are there Broader Benefits of Incredible Years on Children’s Development?

No broader benefits of Incredible Years are found according to parents, teachers, and children: Incredible Years did not reduce children’s peer problems, emotional problems, and hyperactivity 2.5 years after the end of the intervention (Table 1). Similarly, children of parents who participated in Incredible Years did not outperform children in the control condition on the neuropsychological tasks measuring attention and behavior inhibition. Finally, families who participated in Incredible Years did not make less use of services (i.e., special education or post-intervention mental healthcare related to children’s mental health or parenting difficulties) in the years after intervention.

### Post-hoc Analyses

Because children with clinical baseline levels of conduct problems were more likely to have additional mental health problems, we conducted post-hoc analyses to check whether broader benefits of Incredible Years were present specifically in this clinical group. This was not the case. The AN(C)OVAs for the subgroup (*n* = 107) of children with clinical levels of conduct problems did not suggest any significant differences between the control and intervention condition on peer problems, emotional problems, ADHD symptoms and related neurocognitive deficits, and service use. Means, standard deviations, and test results for children with clinical levels of conduct problems are provided in Table 4 of the online supplementary file.

In addition, we conducted post-hoc subgroup analyses to check whether broader benefits of Incredible Years on emotional problems were present specifically in the subgroup of children with elevated baseline emotional problems. This was also not the case. The ANCOVA, controlling for baseline emotional problems, for the subgroup (*n* = 103) of children with clinical levels of conduct problems did not suggest any significant differences between the control and intervention condition on emotional problems (*F*(1,88) = .009, *p* = .976).

Lastly, we conducted moderator analyses of the most often used demographic characteristics (child and parent age and gender, educational level, family composition) and of baseline child (conduct, emotional, and ADHD problems) and parenting measures (negative and positive parenting). In line with moderator analyses on immediate post-intervention levels of conduct problems (Weeland et al. 2017), we found no significant moderation effects on follow-up levels of conduct problems (*p*s > .05).

## Discussion

The wide dissemination of parenting interventions for children’s conduct problems in prevention settings holds the expectation that these interventions prevent children from developing (further) behavior disorders and other mental health problems. Yet, because most evaluation studies have a relatively short timespan and focus on conduct problems only, we know little about the sustained effects of these interventions and their broader effects on other mental health problems. We conducted a longer-term randomized controlled study and used a multi-method and multi-informant approach to estimate the magnitude and width of the longer-term preventive impact of Incredible Years on children’s mental health.

We found significant effects of Incredible Years on parent-reported reduced conduct problems that sustained until the 2.5-year follow-up. The effect size was meaningful, but relatively small, and lower than typically found in indicated prevention trials for children’s conduct problems (*d* = 0.31 as opposed to *d* = 0.55 in Leijten et al. (2019)). Apart from the fact that we examined longer-term, and not direct, effects, this could be related to a relatively low threshold (75th percentile) for family inclusion, which led to the inclusion of families with a wide variety of problem severity ranging from mild to severe. It could also be due to the selective attrition that occurred in our intervention group—families with children with more severe conduct problems were more likely to drop out. Twenty percent of families in the intervention condition did not receive the Incredible Years intervention but were included in the intent-to-treat analyses. A previous analysis showed that families allocated to Incredible Years, but who did not take part in the group meetings, did not significantly improve in disruptive behavior compared to control families (Van Aar et al. 2019). Our intention-to-treat analyses therefore probably yielded conservative effect estimates.

### Sustained Effects of Incredible Years on Conduct Problems

Our finding that reduced parent-perceived conduct problems are maintained until 2.5-year follow-up seemingly contrasts with findings from a previous Incredible Years trial analyzing 10-year follow-up effects (Scott et al. 2014). In this trial, for a clinical treatment sample, sustained effects were observed on children’s conduct problems, but no sustained effects emerged for an indicated prevention sample screened from the general population—similar to the ORCHIDS study. Perhaps the different outcomes can be explained by the fact that the Scott et al. study (Scott et al. 2014) had a smaller sample size, and therefore less statistical power, to detect small effects, and estimated effects across a much longer time interval than we did. Our well-powered, randomized design suggests that families perceived that positive effects on child behavior were maintained until 2.5 years later, suggesting that effects after parenting intervention tend to sustain, rather than accrue or fade away.

The sustained effects of Incredible Years pertained to parent-reported conduct problems specifically; no effects were found using children’s self-reports or teacher reports. This may indicate that children’s conduct problems have changed in interaction with their parents specifically, and not in the school setting that is part of child and teacher report (De Los Reyes et al. 2009). An alternative explanation is that Incredible Years changed parents’ perception of their child’s behavior. A better understanding of their child’s behavior could, for example, lead to parents having more realistic, age-appropriate expectations, and reduced stress, relieving their potentially initial overestimation of their child’s behavior problems (Crnic et al. 2005; Moens et al. 2018). Yet another explanation might be that the questionnaire used by parents (i.e., the ECBI), detected changes in conduct problems that the questionnaire used by teachers and children (i.e., the SDQ), was unable to detect. Although well-established and validated (Bourdon et al. 2005; Stone et al. 2010), the SDQ conduct scale consists of only five items and has only three response options (i.e., not true, somewhat true, and certainly true). This makes the SDQ suitable for screening purposes but perhaps less suitable for assessing children’s behavior changes in the context of a parenting intervention. Moreover, given that the SDQ conduct problems scale also had a lower reliability, the power to detect the already subtle changes was further reduced.

### No Broader Benefits of Incredible Years on Children’s Development

Our finding that there are no broader benefits of Incredible Years on children’s mental health problems shows that reducing coercive parent-child interactions reduces children’s conduct problems specifically. Although coercive interactions and conduct problems often co-occur with other mental health problems (Capaldi 1992; Beauchaine and McNulty 2013), changes in conduct problems do not automatically translate into changes in other mental health problems. Whereas one might suggest that such cascading effects need more time to evolve and become apparent later, studies following children 5 to 10-year post-intervention suggest that this is not the case (Scott et al. 2014; see Sandler et al. 2015 for a review on cascading effects for a broader range of interventions). In situations where the prevention aim is to target a range of mental health problems, transdiagnostic interventions focused on shared underlying mechanisms for different mental health problems may need to be designed (e.g., transdiagnostic interventions; Weisz et al. 2017).

Alternatively, it might be that there are broader benefits of Incredible Years, but that these were too small to detect with our assessment methods. In prevention contexts, problems are generally milder, less developed, and to a lesser extent co-occurring. Higher conduct problem severity is associated with a higher likelihood of co-occurring social-emotional problems (see Overbeek et al. 2001; Sandler et al. 2015), and especially in children characterized by a co-occurrence of externalizing and other problems, some of the broader intervention effects may be found. Another factor that comes into play is that we tend to measure mental health problems as the outcome of preventive effects (e.g., emotional problems and school dropout) rather than the milder forms or antecedents (e.g., negative thoughts and skipping classes at school). This might explain why broader effects are found in treatment setting but not in prevention setting (Scott et al. 2014). More sensitive measures of the antecedents of broader mental health issues may be needed to assess effects of prevention context.

### Strengths and Limitations

Strengths include our randomized controlled design with an intact control condition at 2.5-year post-intervention, our five waves of data, and our relatively large sample of 387 families. We were therefore able to detect even small changes in children’s conduct problems (measured with the ECBI) and to examine whether changes in conduct problems occurred during or after the intervention period. The controlled design appeared essential for estimating true longer-term intervention effects, because conduct problems also reduced in children in the control condition, as part of a naturally occurring developmental trajectory in this age period (Matthys and Lochman 2017). A within-family design, that falsely assumes that without intervention no change would have occurred, would therefore overestimate intervention effects.

Limitations include, first, that conduct problems in the home setting were only assessed by parent-reported questionnaires and were reported by only one parent. Future research should include multiple informants or observations on child behavior at home. Our previously used observations using a 20-min semi-structured play task (Weeland et al. 2017) showed little variance at pretest and were no longer suitable for participating children at an older age. Second, despite baseline equivalence of the core study measures, a lack of baseline values for some of the broader effect measures (i.e., teacher and child reported outcomes) at follow-up complicates an interpretation of the null findings. Third, not all measures were optimally suited to acquire detailed and sensitive measures of post-intervention change. Specifically, we relied on the SDQ to assess teacher- and child-reported conduct problems and wider mental health problems. As discussed before, the SDQ’s limited items and response options may not be sensitive enough to assess subtle changes in children’s mental health. Also, the power to detect subtle changes might have been attenuated due to low reliability of scales, specifically the peer problems scale. Also, our measurement of service use was relatively succinct. Because we do not know to what extent this measure is sensitive to pick up changes in service use in the longer term, the present analysis allows only for a rough estimation of service use in several specific domains.

### Implications and Recommendations

Our study brings several implications for practice. First, Incredible Years is an effective parenting intervention to sustainably reduce parent-perceived conduct problems of children—on average about a third of a standard deviation on the ECBI. Because of the significant overlap between Incredible Years and other “brands” of parenting intervention programs such as Parent Management Training Oregon (PMTO), parent-child interaction therapy (PCIT), or Triple P, for instance, this implies that parenting interventions in general might have sustained effects. Indeed, this conclusion is corroborated by other research showing longer-term intervention effects across different parenting intervention programs (Lundahl et al. 2006; Sandler et al. 2015; Van Aar et al. 2017). Second, based on our finding that conduct problems reduce during the intervention, and not afterward, no further improvements in conduct problems should be expected after the intervention terminates. If conduct problems do not sufficiently reduce during the intervention, this could be a sign that other types of support are needed. Third, based on our findings (but please note the limitations regarding measurements, sample, and design used for this study), no broader longer-term benefits should be expected on social, emotional, and ADHD problems. We therefore suggest that Incredible Years as an indicated prevention should be offered for conduct problems specifically.

Further research could focus on unraveling the meaning of changes in parent-perceived conduct problems, to allow for better predictions of the longer-term cascading effects of the Incredible Years parenting intervention. For example, if it is indeed children’s behavior that changes, rather than just the parent’s perception, this may bring about child-related broader effects (e.g., less peer problems and academic failure). If it is parents’ perceptions of conduct problems that changes, rather than the child’s actual behavior, this may be more related to parental cognitions and bring about more parent-related (vs. child related) broader effects (e.g., less parental stress and more feelings of self-efficacy). Disentangling children’s actual behaviors from parents’ perspectives can, for example, be done by letting parents code videos of child behaviors before and after the intervention. This could shed light on how their perceptions of the severity of disruptive child behavior have changed due to the intervention. In addition to examinations of patterns of change in child and parenting behavior over time, future research is warranted on the question for which families’ parenting interventions are most effective in the long term. More generally, we need more precise estimations of when child behavior, or parents’ perceptions thereof, start to change during the intervention, to enhance our understanding of how interventions work, and to identify at an early stage whether and how much, families will benefit.

## Conclusion

Effects of the Incredible Years parenting intervention on children’s conduct problems are well-sustained according to parents. Two and a half years after the program, parents still perceive less conduct problems in their children. We found no evidence for broader benefits of Incredible Years as a preventive intervention for children’s mental health or on parents’ service use: benefits pertain to parent-reported reduced conduct problems specifically.

## Electronic supplementary material

ESM 1 (DOCX 27 kb)

## Footnotes

1 As can be seen in the figure, data of about 100 families at 1.5-year follow-up were missing. These data were collected but lost due to technical problems at this specific measurement occasion and can be considered as missing completely at random. The present data of this wave showed no oddities: means and standard deviations were in line with other waves. We therefore included the 1.5-year follow-up data for latent growth analysis, and handled missing data using full information maximum likelihood (FIML).
