# Supplementary material for: Longer-Term Outcomes of the Incredible Years Parenting Intervention
Source: Prev Sci. 2020 Oct 27;22(4):419–31. doi: 10.1007/s11121-020-01176-6 (PMC8060237; doi:10.1007/s11121-020-01176-6)
Supplement: Supplementary file 1 — (DOCX 27 kb) [file 11121_2020_1176_MOESM1_ESM.docx]

**Supplementary file**

Table 1

*Differences between Families who Remained in the Study and Families who Dropped Out at 2.5 year Follow-Up*

|  | Families who remained in the study  (*n* = 305) | Families who dropped out (*n =* 82) | *t* or χ^2^  test value | *p-*value |
| --- | --- | --- | --- | --- |
| Condition (control) | 51% | 41% | 2.425 | 0.119 |
| Age child (years) | 6.25 | 6.55 | -1.741 | 0.084 |
| Gender child (girls) | 46% | 41% | 0.442 | 0.506 |
| Age parent (years) | 38.13 | 37.98 | 0.251 | 0.802 |
| Gender parent (female) | 92% | 87% | 0.430 | 0.512 |
| Single parenthood | 9% | 10% | 0.115 | 0.735 |
| Ethnicity (white) | 88% | 83% | 9.981 | 0.076 |
| Educational level (≥ higher vocational) | 52% | 45% | 1.950 | 0.377 |
| Baseline conduct problems (*M*; ECBI) | 132.86 | 134.79 | -0.804 | 0.422 |
| Baseline negative parenting (*M*; PPI) | 2.74 | 2.81 | -0.939 | 0.349 |

*Note.* ECBI = Eyberg Child Behavior Inventory, PPI = Parenting Practices Inventory.

Table 2

*Overview of Timing of Measurements*

|  | Pre-test | Post-test | Follow-Up 1 | Follow-Up 2 | Follow-Up 3 |
| --- | --- | --- | --- | --- | --- |
| Conduct problems (ECBI)  Parent report^a^ | ♦ | ♦ | ♦ | ♦ | ♦ |
| Conduct problems (SDQ) |  |  |  |  |  |
| Parent report^a^ | ♦ |  |  |  | ♦ |
| Teacher report^b^ |  |  |  |  | ♦ |
| Child report^b^ |  |  |  |  | ♦ |
| Peer problems (SDQ) |  |  |  |  |  |
| Parent report^a^ | ♦ |  |  |  | ♦ |
| Teacher report^b^ |  |  |  |  | ♦ |
| Child report^b^ |  |  |  |  | ♦ |
| Emotional symptoms (SDQ) |  |  |  |  |  |
| Parent report^a^ | ♦ |  |  |  | ♦ |
| Teacher report^b^ |  |  |  |  | ♦ |
| Child report^b^ |  |  |  |  | ♦ |
| Hyperactivity (SDQ) |  |  |  |  |  |
| Parent report^a^ | ♦ |  |  |  | ♦ |
| Teacher report^b^ |  |  |  |  | ♦ |
| Child report^b^ |  |  |  |  | ♦ |
| Inhibitory control (SST)^b^ |  |  |  |  | ♦ |
| Attention (SST)^b^ |  |  |  |  | ♦ |
| Help with child rearing^c^ |  |  |  |  | ♦ |
| Special education^c^ |  |  |  |  | ♦ |

*Note.* ECBI = Eyberg Child Behavior Inventory, SDQ = Strengths and Difficulties Questionnaire, SST = Stop Signal Test, performed by child. Follow-Up 1: 4 months post-intervention, Follow-Up 2: 1 year and 6 months post-intervention, Follow-Up 3: 2 years and 6 months post-intervention.

Table 3

*Means, Standard Deviations, and Test Results of Conduct Problems per Wave*

|  | Incredible Years  *M* (*SD*) | Control  *M* (*SD*) | *F* | *p-*value | *d* |
| --- | --- | --- | --- | --- | --- |
| Conduct problems (ECBI) |  |  |  |  |  |
| Pretest | 135.09 (19.82) | 131.40 (18.49) | 3.57 | .060 |  |
| Immediate Posttest | 123.47 (18.04) | 126.52 (19.28) | 11.93 | .001 | 0.35 |
| Follow-up 4 months | 122.50 (19.96) | 122.90 (18.86) | 2.40 | .122 | 0.21 |
| Follow-up 1.5 years | 112.03 (15.23) | 112.42 (16.26) | 1.04 | .310 | 0.21 |
| Follow-up 2.5 years | 114.23 (21.86) | 116.94 (23.93) | 4.69 | .031 | 0.33 |

*Note.* ECBI = Eyberg Child Behavior Inventory. Effect size calculation based on pre-post controlled design, Morris (2008).

Table 4

*Means, Standard Deviations, and Test Results of Broader Mental Health Problems, for the Subgroup of Families (n = 107) with Clinical Levels of Disruptive Behavior*

|  | Incredible Years Mean (*SD*) | | Control  Mean (*SD*) | *F / χ^2^* | *p-*value | *d /* OR^*^ |
| --- | --- | --- | --- | --- | --- | --- |
| Peer problems (SDQ)  Parent report  Teacher report  Child report | | 2.57 (2.01)  1.91 (1.61)  2.39 (1.86) | 2.70 (1.66)  2.33 (2.01)  2.56 (1.80) | 0.18  1.20  0.22 | .667  .278  .642 | 0.07  0.23  0.09 |
| Emotional symptoms (SDQ)  Parent report  Teacher report  Child report | | 4.55 (2.43)  2.74 (1.99)  3.78 (2.28) | 4.28 (2.24)  3.00 (2.20)  3.77 (2.19) | 0.00  0.30  0.00 | .976  .586  .976 | -0.12  0.13  0.00 |
| Hyperactivity (SDQ)  Parent report  Teacher report  Child report | | 6.12 (2.79)  4.88 (2.71)  5.48 (2.57) | 6.76 (2.02)  4.56 (2.50)  5.90 (2.30) | 2.18  0.35  0.72 | .144  .555  .400 | 0.26  -0.12  0.17 |
| Inhibitory control (SST) | | 340.78 (96.42) | 311.01 (98.90) | 2.45 | .121 | 0.31 |
| Attention (SST) | | 672.73 (112.85) | 668.92 (111.32) | 0.03 | .862 | 0.03 |
| Service use  Help with child rearing  Special education | | 63%  24% | 68%  23% | 0.23  0.05 | .631  .817 | 0.80  1.08 |

*Note.* ECBI = Eyberg Child Behavior Inventory, SDQ = Strengths and Difficulties Questionnaire, SST = Stop Signal Test, performed by child.

^*^Effect size calculation based on Cohen’s *d* for continues variables, and Odds Ratio for categorical variables (service use).
